# Valorization of Date Palm Waste for Plastic Reinforcement: Macro and Micromechanics of Flexural Strength

**DOI:** 10.3390/polym13111751

**Published:** 2021-05-27

**Authors:** Chihaoui Belgacem, Ferran Serra-Parareda, Quim Tarrés, Pere Mutjé, Marc Delgado-Aguilar, Sami Boufi

**Affiliations:** 1 Faculty of Science, University of Sfax, LMSE, Sfax BP 802-3018, Tunisia; chihaoui.belgacem@gmail.com (C.B.); Sami.Boufi@fss.rnu.tn (S.B.); 2 LEPAMAP-PRODIS Research Group, University of Girona, Maria Aurèlia Capmany, 61, 17003 Girona, Spain; ferran.serrap@udg.edu (F.S.-P.); pere.mutje@dg.edu (P.M.); m.delgado@udg.edu (M.D.-A.); 3 Chair on Sustainable Industrial Processes, University of Girona, Maria Aurèlia Capmany, 61, 17003 Girona, Spain

**Keywords:** date palm, composites, flexural properties, enzymatic treatment, waste reduction

## Abstract

Date palm waste is an abundant agricultural residue in Tunisia and can be used for plastic reinforcement. Moreover, its use in plastic composites can help to reduce dependence on fossil resources for material production. In this work, the valorization of date palm residues was studied by employing high-yield processes following mechanical, chemical, and enzymatical treatments. Fibers obtained by soft chemical treatment with sodium hydroxide and enzymatic treatment with xylanases and pectinases were evaluated for their use in the reinforcement of plastic materials. The flexural strength property, truly relevant for structural, construction, automotive, or other market sectors, was adopted to assess the reinforcing potential of the fibers. Polypropylene was effectively reinforced with date palm fibers (60 wt.%), exhibiting a flexural strength increases of 80% (73.1 MPa), 93% (78.5 MPa), and 106% (83.9 MPa) for mechanical, chemical, and enzymatic fibers, respectively. The different treatments had an impact on the chemical composition of the fibers, and by extension on the final properties of the composites. The holocellulose content could provide good interfacial adhesion using a coupling agent, whereas the lignin content improved the dispersion of the phases. Two interesting outcomes were that the flexural performance of enzymatic fibers was like that of wood composites, whereas the specific flexural strength was comparable to that of glass fiber composites. Overall, the present work has shown the potential behind date palm waste in the composite sector when a specific property or application is desired. Novel treatments have been used for greater fiber compatibility, increasing the sustainability of the process, and improving the applicability of the palm residue.

## 1. Introduction

The necessity of a significant change in environmental management and the minimization of the impact produced by our society has motivated the development of environmentally sustainable products. In the field of plastic composites, lignocellulosic fibers are viewed as an eco-friendly alternative to non-renewable and non-biodegradable synthetic reinforcements like carbon, aramid, or glass [1,2,3,4]. In addition to its sustainable character, natural fibers have low density, a relatively low cost, and provide reasonable mechanical enhancements [5]. Natural fibers also exhibit good thermal and acoustic insulating characteristics, which could contribute to widening the range of applications of composite materials [6,7]. Indeed, the manufacturing of glass fibers (GFs), the most representative amongst synthetic fibers, is relatively slow, challenging, and may damage the processing equipment. GFs also have the disadvantage of being difficult to dispose of after use [8].

Agricultural residues such as bagasse, rice straw, wheat straw, pineapple leaf, or date palm residues contain high cellulose and hemicellulose contents and therefore low lignin contents [9]. However, agricultural residues are often burned or disposed of in landfills, resulting in environmental pollution. Date palms are spread over vast geographical areas, with the Arab countries having the largest cultivation with more than 100 million palms [10,11]. The date palm is widely cultivated in North and Central Africa for the exploitation of date fruits. However, the large quantities of agricultural residues derived from date harvesting and palm pruning are an important source of biomass. It is estimated that in producing countries such as Tunisia, the annual generation of date palm residues can reach 100 thousand tons [12]. Some research has focused on the valorization of this date palm residue for the production of biochar, bioethanol, or even for water treatment [13,14,15].

**Figure 1:**
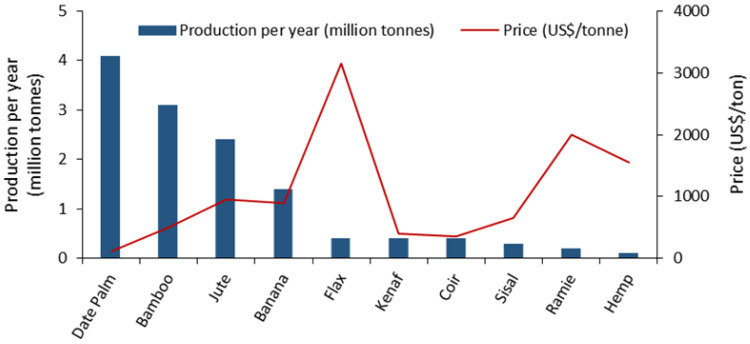
Production per year and price of date palm against other plants [16,17].

**Figure 2:**
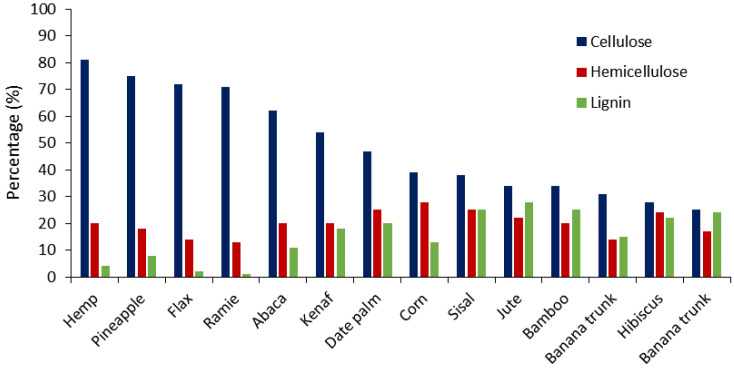
Cellulose, hemicellulose, and lignin content in date palm against other plants [18,19,20].

Sometimes, the residues are used to make animal feed. As shown in Figure 1, date palm residues are abundant and have a lower cost than other commonly used plant fibers in the composite sector, which makes this fiber source an attractive plastic reinforcement from both an economic and environmental viewpoint [16,17]. From a technical perspective, date palm fibers present a relatively high cellulose/lignin ratio, which apparently gives the fibers good reinforcing potential. It is widely known that cellulose is the major crystalline compound in the fiber cell wall that provides the fiber with its strength and stiffness. According to the literature, the cellulose content of date palm fibers is higher than some plant-based fibers such as bamboo, hibiscus, sisal, and jute (Figure 2) [18,19,20].

Lignocellulosic fibers such as coir, hemp, sisal, pineapple fibers, and jute, among others, have been widely used as reinforcement for plastics [21,22,23,24,25]. Recently, the use of residue or waste fibers obtained from a variety of agricultural and forest residues has attracted interest from researchers mainly for sustainability reasons [26,27]. The use of these residues as a fiber source for composite materials could help reduce the impact of deforestation and inspire proper management of such residues, which are usually burned, landfilled, or designated to low value-added applications. Indeed, the similar structure and chemical composition of agricultural residues from plants could make them a competitive alternative for plastic reinforcement [28,29].

Although natural fibers exhibit several benefits as reinforcements, there are some drawbacks associated with their usage. The most relevant is the poor compatibility between both the fibers and the polymer [30]. The hydrophilicity of lignocellulosic fibers prevents spontaneous interactions with the hydrophobic matrix, leading to poor interfacial adhesion. As a result, the stress transfer from the matrix to the reinforcement is not efficient, hindering the increment of the mechanical properties [31]. Hence, strengthening the matrix-fiber adhesion is crucial to optimizing the potential of the lignocellulosic fiber and making it commercially competitive. Obtaining a strong interfacial bonding has been generally pursued in two different ways: (i) fiber treatments [32,33,34,35], and (ii) and use of compatibilizers [36,37,38].

Natural fibers can be treated via mechanical, chemical, and enzymatic procedures [39]. Mechanical treatments break down the fiber cell wall structure via shearing forces to obtain single fibers. These treatments do not significantly change the chemical composition of the raw biomass, and result in fibers with a low length to diameter ratio. This is caused by indistinct fiber breakage in the different regions of the fiber structure. For this reason, mechanically treated fibers usually show a lower reinforcing capacity, which makes this type of fiber a suitable option for less mechanically demanding roles [40]. Advantageously, mechanical treatments are environmentally and economically attractive since the generation of residues is low or even negligible, and no chemicals or enzymes are employed. To improve the performance of mechanical fibers, such fibers can be subjected to the action of chemicals. Chemical treatments usually present low manufacturing yields, thus, the employment of low amounts of chemicals is recommended to avoid the excessive generation of residues and improve the sustainability of the process [41]. One of the most effective and low-cost chemical treatment types is alkaline treatment, which involves the use of sodium hydroxide and anthraquinone as catalyzers [42]. Soft alkaline treatments aim at cleaning the fibers’ surface from some extractives, ashes, and impurities that may hinder the interactions between the polymer and the fibers [43]. Some amounts of lignin and hemicellulose may also be removed during these processes to increase the content of cellulose. Besides, enzymes are much more selective and less aggressive than chemical treatments. Pectinases, xylanases, laccases, and cellulases are the principal enzymes used during enzymatic treatment [44,45]. At an industrial scale, xylanases have attracted attention in various fields such as pulp biobleaching, waste paper recycling, and bioprocessing, among others. The xylanases and pectinases combination is used for extractives removal from the external region of the fiber structure. The action of pectinases improves the accessibility and efficiency of xylanases, leading to efficient extraction of the fibers [46,47]. Overall, the selection of the fiber treatment can vary according to the desired application.

However, fiber treatments still do not solve the issue related to fiber-matrix compatibility. As known, the different chemical structure of hydrophobic polymers and hydrophilic fibers usually makes the incorporation of a compatibilizer necessary to enhance the fiber-matrix adhesion. In the case of polypropylene, which is the most widely used polymer in the composite sector, maleic anhydride polypropylene (MAPP) might improve the fiber-matrix adhesion via two different mechanisms [48]. First, the maleic groups should form covalent bonds through esterification with the available hydroxyl groups on the fiber’s surface. Afterwards, MAPP’s PP chain can diffuse between the unmodified PP chains, forming a physical interaction (entanglement) [36].

It is known that natural fiber composites are employed in sectors such as the automotive and building/construction sectors in products like door panels, headliners, roofing sheets, and windows [49]. Generally, these structure members are required to resist forces that are applied laterally or transversally to their axes, thus being submitted to flexural forces. On the contrary, tensile cases are scarce in comparison, which makes the flexural behavior of these materials relevant to evaluate the potential thereof. Designers are particularly interested in predicting the material’s behavior under bending loads rather than tensile loads [50,51]. The flexural strength of composite materials may also be modeled following micromechanics models to find out the intrinsic flexural strength and reinforcing efficiency of the fibers [52]. To the best of the authors’ knowledge, the intrinsic flexural strength of date palm fibers obtained via different methodologies has not been clearly established. The present study presents a possible route for the valorization of date palm residues. At the same time, it shows the procedure for obtaining a correct interface between the fibers and the matrix in three types of fiber treatment (mechanical, chemical, and enzymatic). Accordingly, the present investigation focuses on the flexural strength of 20–60 wt.% date palm fiber-reinforced polypropylene using a three-point flexural test by submitting the fibers to mechanical, chemical, and enzymatic treatments. Furthermore, the study also explores some relations between the tensile and flexural strengths to predict the intrinsic flexural strength of the fibers.

## 2. Materials and Methods

### 2.1. Materials

Composite materials were prepared using date palm fibers (DPF) as reinforcement and polypropylene (PP) as a plastic matrix. Date palm waste (DPW) was obtained from the annual pruning of date palm trees in the region of Gabes (Tunisia). Isplen PP090 G2M polypropylene was kindly supplied by Repsol Química S.A. (Tarragona, Spain). The density and melt flow index of this polypropylene are 0.905 g/cm^3^ and 35 g/10 min (210 °C; 2.16 kg), respectively. Maleic anhydride polypropylene (MAPP Epolene G-3015) was provided by Eastman Chemical Products (San Roque, Spain) and incorporated into composites as a coupling agent. Enzymes xylanases (Panzea^®^) and pectinases (Pectinex^®^ XXL) were supplied by Novozymes (Bagsværd, Denmark). Reagents for fiber chemical treatment and material characterization were acquired at Sigma-Aldrich and used as received.

### 2.2. Methods

#### 2.2.1. DPF Obtention and Characterization

DPW were initially grounded to reduce the particle size under 3 mm by a blade mill. The particles were then sieved in a 10-mesh screen (2 mm) to eliminate the powder. The resulting material was then passed through a Sprout-Waldron model 105-A defibrillator (Muncy, PA, USA). Fibers obtained from a mechanical defibrillator (DPF-D) were subjected to two different treatments. Then, DPF-D was treated with a 5 wt.% sodium hydroxide (NaOH) solution and 0.1 wt.% anthraquinone (AQ). The process took place in a pressurized reactor at a solid:liquid ratio of 3:7 for two hours at 80 °C. Finally, the fibers were washed to remove excess chemicals with deionized water. The fibers were then submitted to enzymatic treatment with a xylanases and pectinases formulation. The enzyme formulation consisted of xylanases dissolved in phosphate buffer solution (pH7) and pectinases dissolved in acetate buffer solution (pH4.8). Next, the fibers were treated with 1 L of 100 U/g xylanase and 3000 U/g pectinase solution. They were then filtered and washed with deionized water. The chemically and enzymatically treated fibers were labeled DPF-NaOH and DPF-E, respectively. Finally, the obtained fibers were dried in an oven until the achieved a constant weight.

The fibers’ chemical composition was analyzed following Tappi standards. The extractives content was measured according to T 204 cm-97 [53]. The Klason lignin, also referred to as acid-insoluble lignin, was quantified on the extractive-free sample following T 222 om-98 [54]. Parallelly, the ashes content was evaluated following T 211 om-93 [55]. The holocellulose content was determined by difference. The kappa number determination was performed under T 236 cm-85 [56]. A MorFi compact device analyzer (Techpap, Grenoble, France) was used to determine the fiber morphology characteristics (mean fiber length and diameter).

#### 2.2.2. Preparation of Composite Materials and Specimen Obtention

Composite materials were prepared at weight ratios of 80/20, 60/40, and 40/60 (matrix/fiber) using an internal mixer plastograph (Brabender GmbH & Co KG, Duisburg, Germany). Firstly, PP and MAPP were incorporated into the mixing chamber at 180 °C and 80 rpm. When the polymer plasticized, the fibers were incorporated at the above-mentioned ratios. WINMIX software was used to computer-control the mixing process. Then, the compounds were cooled down to room temperature and milled with 10 mm mesh with a Retsch SM 100 knives mill (Retsch Iberia, Llanera, Spain). Then, the resulting material was stored in an oven at 80 °C to prevent moisture absorption.

Normalized flexural and tensile specimens were mold injected by an Arburg 220 M 350-90U (Arburg, Loßburg, Germany) under standard ASTM D3039 [57]. At least 10 specimens were obtained for each formulation. It is worth mentioning that even though the study focuses on the flexural strength of the composites, tensile strength is needed for the application of the micromechanics models and the prediction of intrinsic flexural strength.

#### 2.2.3. Mechanical Properties

The specimens were stored in a climatic chamber at 23 °C and 50% RH before mechanical assessment (ASTM D618 standard [58]). Flexural properties were determined by an Instron™ 1122 universal testing machine (Metrotec S.A., Barcelona, Spain) according to ASTM D790 standard [59]. Moreover, tensile properties were recorded in agreement with ASTM D638 [60].

#### 2.2.4. Density Measurement

The density of the fibers was determined according to Equation (1). From the fibers’ density, the fiber volume fractions were obtained at each fiber content following Equation (2).

(1) $$\rho^{F}\;=\;\frac{w^{F}\rho^{m}}{\frac{\rho^{m}}{\rho^{c}}\cdot \left(w^{m}\;+\;w^{F}\right)\;-\;w^{m}}$$

(2) $$V^{F}\;=\;\frac{w^{F}/\rho^{F}}{w^{F}/\rho^{F}\;+\;w^{m}/\rho^{m}}$$

where $w^{m}$ and $w^{F}$ are matrix and fiber weight fractions, respectively; $\rho^{c}$ and $\rho^{m}$ are the density of the composite and matrix, respectively, both measured employing a pycnometer; and the fiber density and fiber volume fraction are represented by $\rho^{F}$ and $V^{F}$, respectively.

#### 2.2.5. Micromechanics of the Flexural Strength

The modified Rule of Mixtures (mRoM) is a simple and effective model for matrix and fiber contribution to the composite strength computation. The modified Rule of Mixtures simplifies the composite’s strength as the matrix contribution and the stress transfer through the reinforcement. This equation was initially conceived for tensile property prediction, but it may also be used to determine flexural properties. The mRoM for flexural and tensile strength is presented in Equations (3) and (4), respectively.

(3) $$\sigma_{f}^{c}\;=\;f_{c,f}\cdot \sigma_{f}^{F}\cdot V^{F}\;+\;\sigma_{f}^{m^{*}}*\left(1\;-\;V^{F}\right)$$

(4) $$\sigma_{t}^{c}\;=\;f_{c,t}\cdot \sigma_{t}^{F}\cdot V^{F}\;+\;\sigma_{t}^{m^{*}}*\left(1\;-\;V^{F}\right)$$

The matrix contribution to the tensile and flexural strength of the composites is represented by $\sigma_{t}^{m^{*}}$ and $\sigma_{f}^{m^{*}}$. These values are obtained from the matrix stress-strain curve at the maximum deformation of the composite. $\sigma_{f}^{c}$ and $\sigma_{t}^{c}$ are the flexural and tensile composite strength. The tensile and flexural coupling factors were represented by $f_{c,t}$ and $f_{c,f}$. The coupling factors are incorporated into the mRoM to correct the contribution of the fibers to the composite strength and the intrinsic tensile and flexural fiber strength were $\sigma_{t}^{F}$ and $\sigma_{f}^{F}$, respectively.

The experimental measurement of the fibers’ intrinsic properties is difficult due to short fiber lengths. In this sense, each equation contains two unknowns, the intrinsic fiber strength, and coupling factor. Through the Kelly and Tyson modified equation and the Bowyer and Bader solution, the intrinsic tensile strength of fiber may be calculated [61,62]. Generally applied to glass fiber composites, the values obtained from the mathematical models showed good agreement with the experimental values determined through typical single fiber analysis. The models are described in detail in previous works [63,64]. Knowing the intrinsic tensile strength, it is possible to obtain the tensile coupling factor from Equation (4).

On the other hand, the intrinsic fibers’ flexural strength is not simple to determine. However, a linear relationship between the macro and micromechanical properties of composite materials was proposed by Hashemi. This relationship can be defined by the following equation: $\sigma_{f}^{F}\;=\;\left(\sigma_{f}^{c}/\sigma_{t}^{c}\right)\cdot \sigma_{t}^{F}$ [65]. Though, as the same author warned, this assumption may not be appropriate for all types of composites. Instead, an alternative methodology was developed to obtain the intrinsic flexural strength. As is well known, fiber morphology, efficiency, and orientation influence the coupling factor, contrary to the type of test performed. Thus, it is assumed that the magnitude order of flexural and tensile coupling factors are the same $\left(f_{c,f}\;\equiv \;f_{c,t}\right)$ [66]. In this context, the tensile strength contribution and the flexural strength contribution of the reinforcement are comparable to the fiber intrinsic strengths.

The fibers’ contribution to the composite strength can be determined by the mRoM. The representation of $\sigma_{f}^{c}\;-\;\sigma_{f}^{m^{*}}\cdot \left(1\;-\;V^{f}\right)$ and the fiber volume fraction $\left(V^{F}\right)$, results in the mean fibers contribution to the material strength as the slope of the plotted line. The obtained factors are the fiber tensile strength factor (FTSF) and the fiber flexural strength factor (FFSF), as previously reported by Thomason (Thomason, 2002).

(5) $$\mathrm{FFSF}\;=\;f_{c,f}\cdot \sigma_{f}^{F}\;=\;\left(\frac{\sigma_{f}^{c}\;-\;\sigma_{f}^{m^{*}}*\left(1\;-\;V^{F}\right)}{V^{F}}\right)$$

(6) $$\mathrm{FTSF}\;=\;f_{c,t}\cdot \sigma_{t}^{F}\;=\;\left(\frac{\sigma_{t}^{c}\;-\;\sigma_{t}^{m^{*}}*\left(1\;-\;V^{F}\right)}{V^{F}}\right)$$

The intrinsic fiber flexural strength can be obtained by Equation (7), using the values of the tensile and flexural strength contribution of the fibers, and its intrinsic tensile strength.

(7) $$\frac{\sigma_{f}^{F}}{\sigma_{t}^{F}}\;=\;\frac{\mathrm{FFSF}}{\mathrm{FTSF}}$$

**Figure 3:**
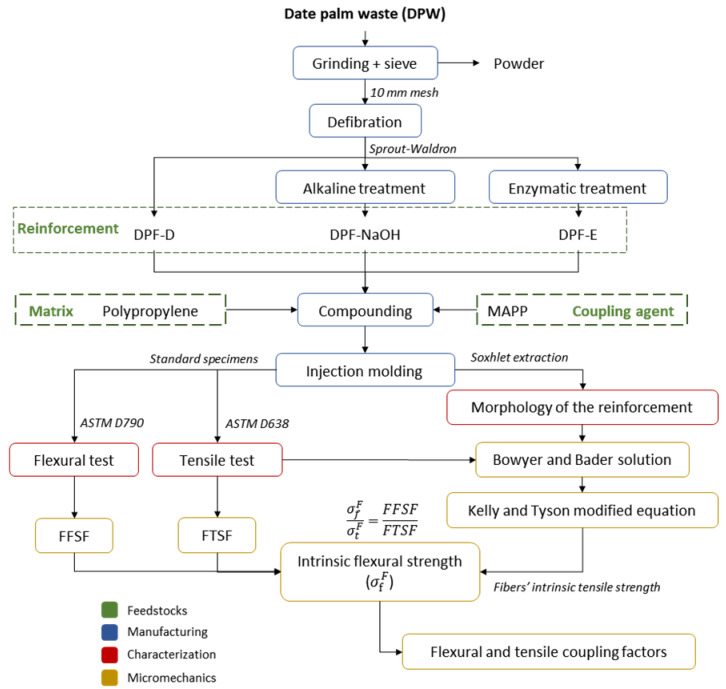
Workflow of the present investigation.

Towards a better understanding of the following methodology, Figure 3 illustrates the experimental workflow of the investigation, which goes from raw materials to micromechanics models.

## 3. Results

### 3.1. Chemical Composition of the Fibers

The chemical composition of the different obtained fibers (DPF-D, DPF-NaOH, and DPF-E) was analyzed as an important parameter affecting the mechanical performance of the fibers. Table 1 presents the content of holocellulose, Klason lignin, ashes, and extractives of the fibers, as well as the kappa number. The process yield is also presented as an indicator of the treatment intensity.

DPF-D presented the highest content of lignin (26.5 wt.%), extractives (2.6 wt.%), and ashes (7.4 wt.%) while presenting a relatively low content of holocellulose (63.5 wt.%). Such percentages were partly expected since cold defibration processes do not significantly modify the chemical composition of the raw biomass, apart from dissolving some cold-soluble extractives and removing minor quantities of ashes. Indeed, the process yield was high with a value of 99%, suggesting the preservation of chemical constituents. The submission of the fibers to a 5 wt.% of NaOH decreased the lignin content from 26.5 to 24.6 wt.%; accordingly, the kappa number was also reduced from 87.5 to 66.6. The lignin removal was not excessive since the severity of the treatment was not high and only 14% of the raw material was not retained. Similar effects on the lignin content of natural fibers are observed in the literature after a 5 wt.% NaOH addition [32]. The alkaline treatment also removed part of the ashes and extractives, finally incrementing the holocellulose content to 67.7 wt.%. Chemical composition results are shown according to the fiber structure modification reported in the literature [41]. As expected, the action of the chemical pretreatment causes a reduction of the crystallinity of the fiber while increasing the internal surface area due to a fiber swelling effect. The enzymatic treatment had a more severe effect on the lignin, extractives, and ash content, reaching a process yield of 83%. Thus, the holocellulose content was noticeably increased to a value of 77.4 wt.%. The similar process yields obtained via the chemical and enzymatical route indicate similar treatment intensities and make the performance of the fibers in composites comparable.

**Figure 4:**
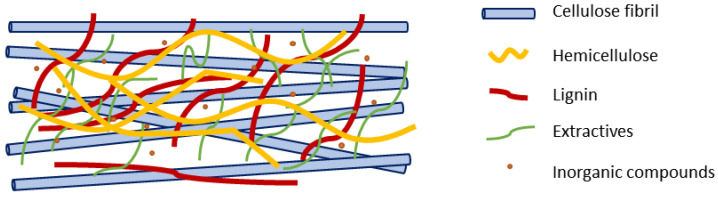
Illustration of the main chemical constituents in the fiber cell wall.

Even though it is true that no enzyme was tasked with the removal of ashes and lignin, the degradation of pectins and xylans may benefit from the release of other chemical constituents. The action of enzymes is mainly focused on the external zone of fibers, where the concentration of lignin, extractives, and ashes is high [67]. In these layers, cellulose, hemicellulose (xylans), lignin, pectins, and inorganic compounds form a resistant and functional complex, as illustrated Figure 4. Hence, the breakdown of any of these constituents may inevitably remove the attached chemical constituents. Enzymatic treatment, on the other hand, leads to a reduction of fibers and the formation of sugars.

Some authors also suggest the existence of strong binding between the carbohydrate molecules to lignin, favoring the presence of lignin-carbohydrate complexes (LCC) [68]. Xylans and pectins are known to bind lignin preferentially, being only weakly associated with cellulose [69]. Hence, the breakdown of xylans and pectins may promote lignin removal, with cellulose being affected to a lesser extent. Overall, the maintenance of cellulose is interesting since it provides strength and stiffness to the fiber. Moreover, the lignin and extractives removal allows for the presence of free hydroxyl groups at the fiber surface, which could finally extend the reaction between the hydroxyl groups and maleic anhydride rings of the coupling agent.

### 3.2. Coupling Agent Optimization

**Figure 5:**
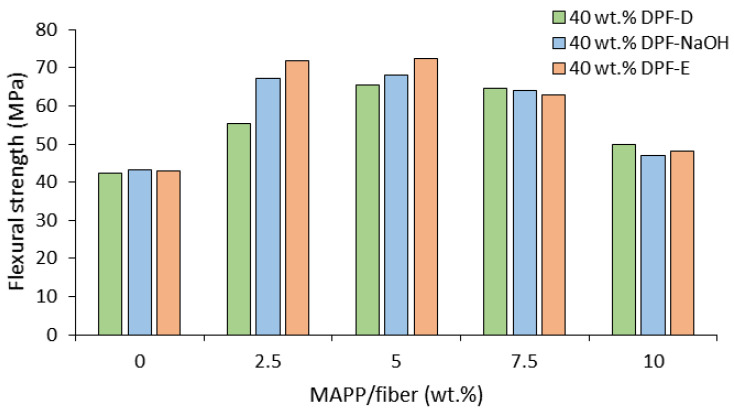
Effect of coupling agent concentration on composites loaded with a 40 wt.% of date palm fibers.

The composite flexural strength is a function of the fiber resource and amount, its morphology and orientation, the dispersion and distribution of the fiber inside the matrix, and mainly the fiber-matrix adhesion. The interfacial adhesion may be enhanced by the addition of maleic anhydride polypropylene (MAPP), which promotes interaction between the fibers and the polymer. Hence, the present work explored the effect of different MAPP concentrations on the flexural strength of the composites. The coupling agent was added to composites reinforced with 40% DPF-D, DPF-NaOH, and DPF-E at concentrations of 0, 2.5, 5, 7.5, and 10 wt.% of the fiber content. It was assumed that the optimal concentration of MAPP would be found when the flexural strength of the composites was higher. The effect of MAPP on the flexural strength of the composites is presented in Figure 5.

The addition of a 5 wt.% of MAPP yielded the highest flexural strength values, suggesting the enhancement of the interfacial adhesion. Composites without MAPP showed a similar flexural strength to polypropylene (40.6 MPa), suggesting poor fiber-matrix compatibility. The flexural strength decreased at MAPP contents below 5 wt.%, whereas at contents over 5 wt.% the flexural strength decreased again. The flexural strength decrease at higher MAPP concentrations can be ascribed to self-entanglements and slippage between MAPP chains. It is worth mentioning that composites loaded with DPF-NaOH and DPF-E showed little difference between the 2.5 and 5.0 wt.% of MAPP. This is attributed to the higher hydroxyl group availability in these fibers given by the higher holocellulose content, allowing for a higher capacity of MAPP-fiber interaction at lower MAPP concentrations.

### 3.3. Flexural Properties of the Composites

PP was loaded with a 20, 40, and 60 wt.% of DPF-D, DPF-NaOH, and DPF-E. The flexural properties of date palm composites are presented in Table 2. $V^{F}$ is the volume fraction of fiber, $\sigma_{f}^{c}$ is the composite flexural strength, $\varepsilon_{f}^{c}$ is the maximum flexural deformation, and $\sigma_{f}^{m^{*}}$ is the matrix contribution to the composite flexural strength. Using the density of polypropylene (0.905 g/cm^3^), DPF-D (1.31 g/cm^3^), DPF-NaOH (1.37 g/cm^3^), and DPF-E (1.47 g/cm^3^), the fiber volume fraction was obtained. The matrix contribution values were obtained from flexural stress-deformation curves of the PP, taking the strength value of the matrix at the maximum composite deformation.

The flexural strength of the composites increased with the fiber content regardless of the DPF type. The achievement of good dispersion and distribution of fibers inside the matrix can be demonstrated by the linear increase of the flexural strength versus the fiber volume fraction. Indeed, polypropylene could be loaded up to 60 wt.% of reinforcement without decreasing the flexural strength. The fact that polymers can withstand high reinforcement contents makes possible the use of less matrix while maintaining the mechanical properties of composites. This is an important economic and also environmental advantage. Contrarily, highly delignified fibers tend to form aggregates at elevated contents due to the great number of hydroxyl groups. Recent work highlighted the importance of lignin in providing a good dispersion and distribution of the fibers throughout the matrix [70]. As reported in this work, reducing the lignin content below the 8 wt.% could directly affect the stress-transfer quality between the fibers and the matrix, and thus decrease the mechanical property. Furthermore, other authors review the use of lignin as a binder between fiber and plastics [71,72]. Generally, an equilibrium between the holocellulose content, which ensures a good interfacial adhesion by interacting with the coupling agent, and the lignin content, which gives a good fiber dispersion, is necessary to obtain the optimal mechanical properties of natural fiber composites.

Overall, at 60 wt.% of reinforcement, the flexural strength incremented by 80%, 93%, and 106% for mechanical, chemical, and enzymatic treated fibers, respectively. These significant increases are comparable to those obtained for other lignocellulosic resources from wood, since it is an agricultural residue with low added value [73,74]. A previous work where wood fibers were used as polypropylene reinforcement returned 75 MPa at 40 wt.% and 80 MPa at 50 wt.% of fiber content. Besides, annual plants, which usually present higher holocellulose contents and aspect ratios, displayed 87 MPa and 103 MPa at 40 and 50 wt.% of reinforcement content.

**Figure 6:**
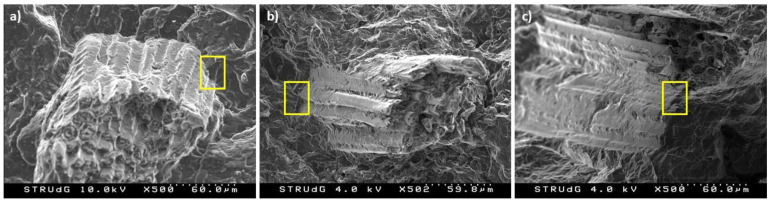
SEM micrograph of the flexural fractured surface for (**a**) mechanical pretreated date palm fibers; (**b**) chemical pretreated date palm fibers; (**c**) enzymatic pretreated date palm fibers.

**Figure 7:**
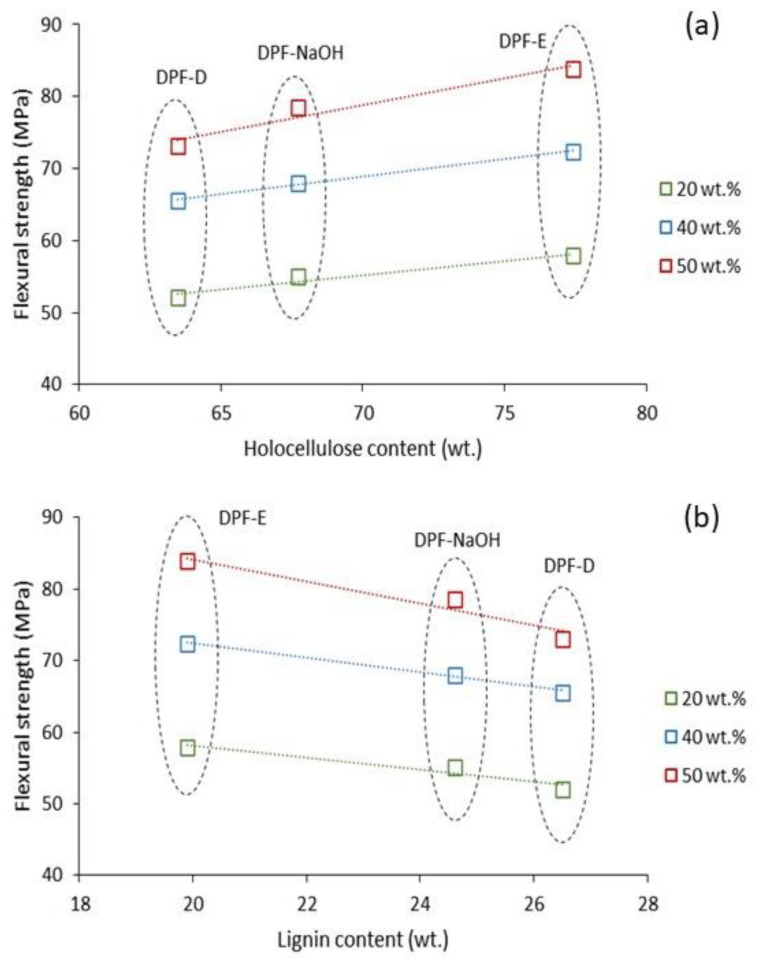
Evolution of the flexural strength of composites with the holocellulose (**a**) and lignin content (**b**).

Within the different types of fibers, DPF-D showed lower reinforcing capacity than DPF-NaOH and DPF-E. As mentioned before, this could be attributed to the effect of the treatment on the chemical composition of the fibers. The removal of lignin and extractives in DPF-NaOH and DPF-E increased the holocellulose, providing the fiber greater rigidity and strength, as well as a higher capacity of forming linkages with the matrix using the coupling agent. The SEM micrograph (Figure 6) showed the improved interface of DPF-NaOH and DPF-E composites. This interface could be related to the high superficial hydroxyl groups available to be linked with the anhydride maleic group of coupling agent. Thereby, apparently, one can expect that the flexural strength of the composites will increase with the holocellulose content, whereas the higher presence of lignin will harm the property. Figure 7 represents the holocellulose and lignin content of DPF-D, DPF-NaOH, and DPF-E against the composite’s flexural strength.

A linear correlation between the flexural strength of the composites and holocellulose/lignin content of the fibers can be found. As mentioned before, such linearity may not be extrapolated to fibers submitted to severer treatments where the low lignin content can negatively affect the mechanical properties of the composite.

The morphological characteristics also influence the mechanical performance of the fibers and such characteristics are highly affected by the treatment applied. To this end, the fibers were extracted from the composites via Soxhlet extraction, and their morphology was determined (Table 3).

It is observed that the submission of the fibers to NaOH and enzymes decreased both the mean fiber length and diameter. Such reduction was more severe in DPF-E, where the diameter reached values around 18 µm. Despite considerable changes in the morphology, the aspect ratio of the fibers remained almost constant within the different types of fibers, which indicates a similar reinforcing potential in terms of morphology. Thereby, the role of chemical composition on the fibers’ reinforcing capacity is highlighted, as well as the linearity observed in Figure 7. Another important outcome from fiber morphology was the reduction of the fibers’ length as the fiber content increased. This is explained by an increment of the blend viscosity caused by the fiber content, which increments the shearing forces during the mixing process and leads to fiber attrition.

From Table 2, it is possible to observe that the deformation of the composites was considerably reduced by the addition of DPF. This was expected, since natural fibers are stiffer than thermoplastics, and thus, its addition contributes to the brittleness of the composite. The effect of fiber treatment on the maximum deformation of the composites was not clear. ANOVA analysis at a 5% confidence rate revealed no significant differences between the deformation capacities of the composites regarding the fiber treatment. However, the same statistical analysis revealed significant differences between the flexural strength of the composites depending on the treatment applied.

One of the objectives of using natural fibers is the displacement of conventional synthetic reinforcements, with glass fibers (GF) being the most common representative of this group. The competitiveness of the developed composite materials reinforced with DPF was evaluated by comparing with previous work results using GF-sized PP composites [75]. Similar flexural strength values were observed comparing composites reinforced with 40% DPF and 20% GF, as well as with 60% of DPF and 30% of GF. However, when it comes to specific properties, the low density of natural fibers makes the specific flexural strength of the composites comparable at the same fiber contents. Hence, DPF shows good competitiveness with GF at a lower cost and material weight.

### 3.4. Intrinsic Flexural Strength of DPF

As mentioned during the methods section, the calculus of the intrinsic flexural modulus requires the measurement of the tensile strength of the composites, which are reported in Table 4.

**Figure 8:**
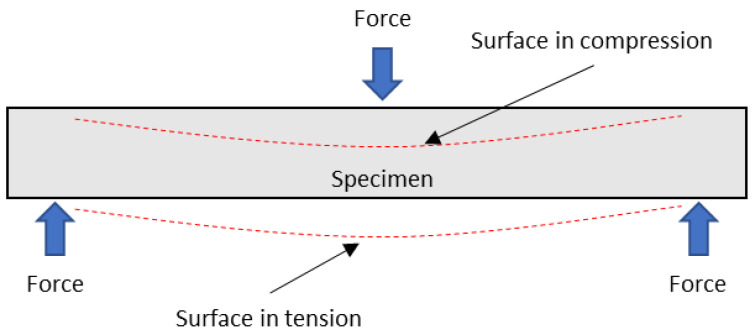
Compression and tensile force combination during the flexural test.

The tensile strength of the specimens showed similar behavior to the flexural strength. Nonetheless, flexural strength values were higher than tensile ones. This is attributed to the combination of the tensile and compressions forces applied to the specimens during the three-point bending test, as illustrated in Figure 8.

Polypropylene, like most polyolefins, can withstand higher loads at compression rather than tensile. Thus, the specimen zone where compression forces act is expected to contribute more to the strength of the material than the one under tensile. Therefore, materials will withstand higher loads when submitted to flexural forces rather than tensile ones.

The anisotropy behavior of natural fiber composites and fiber semi-alignment inside the matrix contribute to the flexural strength. Indeed, it was observed that the fibers’ contribution to the composite flexural strength was higher than to the tensile strength.

**Figure 9:**
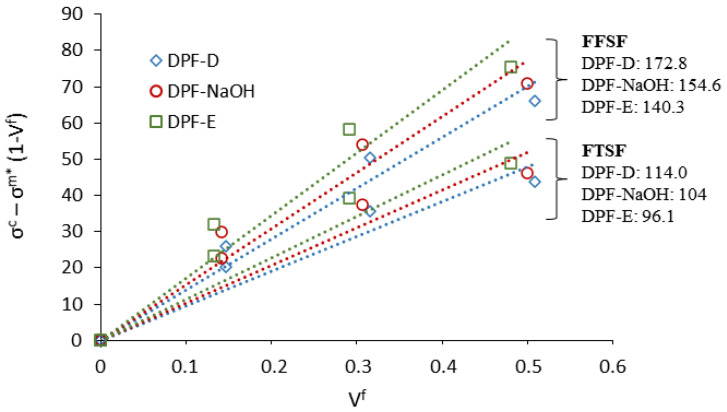
Fiber tensile strength factor and fiber flexural strength factor of date palm fiber composites.

The FFSF was 127.8, 154.6, and 140.3, and the FTSF was 114.0, 104.0, and 96.1 for composites with mechanical, chemical, and enzymatic treated fibers, respectively (Figure 9). As expected, the FFSF exhibited higher values than the FTSF, due to the larger contribution of the fibers to the flexural strength. The ratios between the FFSF and FTSF were set at 1.51, 1.49, and 1.46, respectively. In accordance with Hashemi [65], the contribution of the fibers to the composite flexural strength is greater than to the tensile strength by as much as 1.50 times. In this work, Hashemi dealt with glass fiber composites, though the approximation is not far from the results obtained with date palm fibers.

In previous work, we were able to determine the average intrinsic tensile strength (σ_f_^F^) of DPF-D, DPF-NaOH, and DPF-E as 541 MPa, 607 MPa, and 719 MPa, respectively. In this work, the intrinsic tensile strength of the fibers was computed via the Kelly and Tyson modified equation [61] and its solution provided by Bowyer and Bader [62]. Such models have been applied in a variety of works and have offered an effective prediction of the fibers’ intrinsic tensile strength [76,77]. Considering the FFSF/FTSF ratio, the mean intrinsic flexural strength of the fibers was computed according to Equation (7). From the intrinsic tensile and flexural fiber strength, it was possible to calculate both tensile and flexural coupling factors (Table 5) by means of the mRoM (Equations (3) and (4)).

The intrinsic flexural strength of the reinforcing fibers was found to be 817 MPa, 904 MPa, and 1050 MPa for DPF-D, DPF-NaOH, and DPF-E, respectively. The intrinsic flexural strength of DPF-D and DPF-NaOH was in line with other agricultural waste [78]. However, the value of DPF-E was in the range of 1000–1200 MPa, which is the intrinsic flexural strength for wood fibers as a reinforcement for PP, thus, the potential of enzymatically treated fibers as a replacement to wood fibers is highlighted. The coupling factors were around 0.2, which is inside the range of composites with good fiber-matrix compatibility.

### 3.5. Techno-Economic Challenges and Research Perspectives

Biocomposites offer the possibility of producing products with the right properties for a range of applications [79,80], and represent an important environmental improvement [81]. The use of biocomposites based on natural fibers presents itself as an opportunity for application in the automotive industry, where lightweight construction is an important factor. Nowadays, automotive companies are trying to reduce the use of synthetic fibers by adding natural fibers to the non-structural plastic parts of vehicles. The ecological awareness of today’s society and the incipient legislative actions of governments promote the study and use of bio-based materials.

However, biocomposites based on natural fibers and conventional plastic polymers do not completely solve the environmental problems associated with the use of fossil-based materials such as polypropylene. Typically, the content of natural fibers ranges from 10 to 50 wt% [82]. Therefore, at least 50 wt.% of biocomposite material is based on fossil resources. The use of bio-based plastics (bioplastics) could potentially contribute to improving the environmental performance of future plastic products. Specifically, bio-based polyethylene (BioPE) is industrially available and can be manufactured from biomass, e.g., sugarcane [83]. Today, the production of bioplastics is relatively expensive [84]. Therefore, the addition of natural fibers would be very beneficial from the point of view of cost and mechanical properties.

In this regard, biocomposites of bio-based matrices with similar natural fibers are emerging as new materials [85]. The most plausible alternative to these conventional composite materials focuses on the substitution of both the polymeric matrix and the fibrous reinforcement by components from renewable and sustainable sources, which allow a higher degree of environmental sustainability. In this line, there are many research efforts developed for the incorporation of more environmentally friendly fibrous reinforcements, and that on the other hand allow us to achieve properties corresponding to those requested for application.

In this sense, we can currently find matrices from renewable and biodegradable sources in controlled media, such as the lactic polyacid (PLA), which is the great representative of this group. PLA is a synthetic biopolymer, whose monomer is obtained industrially from the fermentation processes of corn or sugar cane starch. It has good basic mechanical strength (40–60 MPa), good processability, and low water absorption capacity. On the other hand, it has low deformability, although this can be remedied by incorporating plasticizing agents. PLA is one of the current polymers of the biodegradable and renewable variety with the greatest global projection, with a market economic perspective of more than 4.5 billion € in 2020, and a compound annual growth rate of more than 19.5% (CAGR) for the period from 2013–2020. The expected PLA production in 2020 is expected to be 7 times higher than in 2012, reaching absolute values above 900 million tons. In comparative terms, the annual growth of glass fiber reinforced composites is expected to be around 4% per year for the same period, a fact that demonstrates the prospects for growth and expansion of PLA in the medium to long term.

It is also worth mentioning, as a negative factor, the higher production price of PLA compared to, for example, PP, although the economic gap is becoming narrower. In this line, the incorporation of components that lower the product price and also provide improvements to the material is one of the current ways of optimization. Currently, work has already been carried out on the successful incorporation of natural fibers into a PLA matrix. However, the interaction between the PLA matrix and the lignocellulosic fibers still presents a major challenge for researchers. There are several parameters that, a priori, do not allow establishing a common trend and interpretation in the reinforcement of PLA with cellulosic fibers. On the one hand, the matrix itself, since from a commercial point of view there are polymers with great variability of base mechanical strength, ranging from 40 to 110 MPa. On the other hand, the nature of the fiber itself and the content of lignin and other extractives of the virgin fiber, which condition the existence of free hydroxyl groups on the surface of the reinforcement that makes interaction with the matrix very difficult. And finally, the degree of fiber dispersion in the matrix, which controls the optimum fiber-matrix stress transfer.

## 4. Conclusions

Date palm fibers were obtained via mechanical, chemical, and enzymatic processes from the original waste and incorporated as a reinforcement for polypropylene. The effect of fiber treatment on the flexural strength of the composites was studied. Flexural strength is considered a useful property in the structural, construction, automotive, and other market sectors to assess the viability of the material. The flexural behavior was investigated from a macro and micromechanical viewpoint.

It was found that with the addition of 5 wt.% of MAPP to fiber content, the efficiency of date palm fibers as reinforcing agents was enhanced. The presence of this coupling agent allows the use of a higher content of natural fibers and therefore a reduction in the content of material of fossil origin used. This leads to technically competitive and environmentally friendly materials. Fibers obtained through enzymatic treatment showed higher strength, followed by chemical and mechanical fibers. The differences in the flexural strength were attributed to the chemical composition of the fibers, which played a key role in providing an optimal stress transfer between the phases. The flexural strength of composites reinforced with DPF-E were similar to wood-composites, whereas the specific flexural properties were comparable to those of glass fiber-composites.

In the micromechanical analysis, the intrinsic flexural strength of the fibers was established, obtaining a higher value in enzymatic fibers (1050 MPa). This value was found to be in line with wood fibers, thus confirming the great potential of date palm waste in the substitution of wood in polymer composites. The flexural coupling factors were high, confirming good compatibility between the phases.

## Figures and Tables

**Table 1:** Date palm fiber chemical composition.

Sample	Ashes (wt.%)	Extractives (wt.%)	Klason Lignin (wt.%)	Kappa Number	Holocellulose (wt.%)	Process Yield (%)
DPF-D	7.41 ± 0.16	2.59 ± 0.12	26.5 ± 0.31	87.5	63.5	99
DPF-NaOH	5.70 ± 0.24	1.98 ± 0.08	24.6 ± 0.22	66.6	67.7	86
DPF-E	1.97 ± 0.14	0.72 ± 0.07	19.9 ± 0.17	43.9	77.4	83

**Table 2:** Flexural properties of date palm fiber-based composites at 20, 40, and 60 wt.% fiber content.

Fiber	Fiber Content (wt.%)	V^F^	σ_f_^c^ (MPa)	ε_f_^c^ (%)	σ_f_^m^ * (MPa)
	-	-	40.6 ± 0.7	10.8 ± 0.9	40.6 ± 0.6
DPF-D	20	0.147	52.1 ± 1.1	4.8 ± 0.7	30.7 ± 1.0
	40	0.315	65.6 ± 0.9	3.0 ± 0.8	22.0 ± 1.2
	60	0.509	73.1 ± 0.7	1.9 ± 0.6	14.5 ± 0.9
DPF-NaOH	20	0.142	55.1 ± 0.5	4.3 ± 0.4	29.2 ± 0.6
	40	0.307	68.0 ± 1.0	2.8 ± 0.6	20.3 ± 0.8
	60	0.499	78.5 ± 1.1	2.0 ± 0.5	15.2 ± 0.8
DPF-E	20	0.133	57.9 ± 0.6	4.3 ± 0.4	29.9 ± 0.5
	40	0.291	72.4 ± 1.2	2.7 ± 0.3	19.8 ± 0.4
	60	0.480	83.9 ± 0.4	2.1 ± 0.7	16.2 ± 0.9

**Table 3:** Mean fiber length and diameter of extracted date palm fibers.

Fiber	Reinforcement (wt.%)	l_ww_^f^ (µm)	d^f^ (µm)	Aspect Ratio (l_ww_^f^/d^f^)
Extracted DPF-D	20	721.6	29.7	24.3
	40	682.0	29.8	22.9
	60	593.0	29.8	19.9
Extracted DPF-NaOH	20	662.9	27.4	24.2
	40	650.1	27.4	23.7
	60	568.5	27.6	20.6
Extracted DPF-E	20	462.6	18.3	25.3
	40	434.5	18.0	24.1
	60	358.2	18.2	19.7

**Table 4:** Tensile strength of composite and matrix contribution to the tensile strength of date palm fiber composites.

Fiber	Reinforcement (wt.%)	V^f^	σ_t_^c^ (MPa)	σ_t_^m^* (MPa)
-	-	-	27.6 ± 0.1	27.6 ± 0.1
DPF-D	20	0.147	37.6 ± 0.3	20.3 ± 0.4
	40	0.315	47.2 ± 0.2	17.0 ± 0.2
	60	0.509	51.9 ± 0.4	16.4 ± 0.5
DPF-NaOH	20	0.142	39.6 ± 0.3	19.9 ± 0.2
	40	0.307	49.5 ± 0.2	17.3 ± 0.3
	60	0.499	54.8 ± 0.5	17.2 ± 0.5
DPF-E	20	0.133	42.0 ± 0.4	21.5 ± 0.3
	40	0.291	52.3 ± 0.2	18.6 ± 0.2
	60	0.480	58.3 ± 0.4	18.2 ± 0.4

**Table 5:** Mean intrinsic tensile fiber strength, flexural strength, and flexural and tensile coupling factors of the fibers.

Sample	Flexural		Tensile	
	σ_f_^F^ (MPa)	fc,f	σ_t_^F^ (MPa)	fc,t
DPF-D	817	0.19	541	0.20
DPF-NaOH	904	0.20	607	0.20
DPF-E	1050	0.19	719	0.18

## Data Availability

The data presented in this study are available on request from the corresponding author.
